# Nucleobase-Derived Nitrones: Synthesis and Antioxidant and Neuroprotective Activities in an In Vitro Model of Ischemia–Reperfusion

**DOI:** 10.3390/ijms23063411

**Published:** 2022-03-21

**Authors:** Beatriz Chamorro, Iwona E. Głowacka, Joanna Gotkowska, Rafał Gulej, Dimitra Hadjipavlou-Litina, Francisco López-Muñoz, José Marco-Contelles, Dorota G. Piotrowska, María Jesús Oset-Gasque

**Affiliations:** 1 Department of Biochemistry and Molecular Biology, Faculty of Pharmacy, Complutense University of Madrid, Plaza Ramón y Cajal s/n, Ciudad Universitaria, 28040 Madrid, Spain; beatrcha@ucm.es; 2 Faculty of Health, Camilo José Cela University, Castillo de Alarcón 49, Villanueva de la Cañada, 28692 Madrid, Spain; flopez@ucjc.edu; 3 Bioorganic Chemistry Laboratory, Faculty of Pharmacy, Medical University of Lodz, Muszyńskiego 1, 90-151 Lodz, Poland; iwona.glowacka@umed.lodz.pl (I.E.G.); joanna.gotkowska@umed.lodz.pl (J.G.); rafalgulej@gmail.com (R.G.); 4 Department of Pharmaceutical Chemistry, School of Pharmacy, Faculty of Health Sciences, Aristotle University of Thessaloniki, 54124 Thessaloniki, Greece; hadjipav@pharm.auth.gr; 5 Neuropsychopharmacology Unit, “Hospital 12 de Octubre” Research Institute, 28041 Madrid, Spain; 6 Laboratory of Medicinal Chemistry, Institute of Organic Chemistry (CSIC), Juan de la Cierva 3, 28006 Madrid, Spain; jlmarco@iqog.csic.es; 7 Instituto de Investigación en Neuroquímica, Universidad Complutense de Madrid, Ciudad Universitaria, 28040 Madrid, Spain

**Keywords:** antioxidants, neuroprotection, necrosis, apoptosis, oxidative stress, nucleobase-derived nitrones, brain ischemia

## Abstract

Herein, we report the synthesis, antioxidant, and neuroprotective properties of some nucleobase-derived nitrones named **9a**–**i**. The neuroprotective properties of nitrones, **9a**–**i**, were measured against an oxygen-glucose-deprivation in vitro ischemia model using human neuroblastoma SH-SY5Y cells. Our results indicate that nitrones, **9a**–**i**, have better neuroprotective and antioxidant properties than α-phenyl-N-tert-butylnitrone (**PBN**) and are similar to N-acetyl-L-cysteine (**NAC**), a well-known antioxidant and neuroprotective agent. The nitrones with the highest neuroprotective capacity were those containing purine nucleobases (nitrones **9f**, **g**, B = adenine, theophylline), followed by nitrones with pyrimidine nucleobases with H or F substituents at the C5 position (nitrones **9a**, **c**). All of these possess EC_50_ values in the range of 1–6 μM and maximal activities higher than 100%. However, the introduction of a methyl substituent (nitrone **9b**, B = thymine) or hard halogen substituents such as Br and Cl (nitrones **9d**, **e**, B = 5-Br and 5-Cl uracil, respectively) worsens the neuroprotective activity of the nitrone with uracil as the nucleobase (**9a**). The effects on overall metabolic cell capacity were confirmed by results on the high anti-necrotic (EC_50′_s ≈ 2–4 μM) and antioxidant (EC_50′_s ≈ 0.4–3.5 μM) activities of these compounds on superoxide radical production. In general, all tested nitrones were excellent inhibitors of superoxide radical production in cultured neuroblastoma cells, as well as potent hydroxyl radical scavengers that inhibit in vitro lipid peroxidation, particularly, **9c**, **f**, **g**, presenting the highest lipoxygenase inhibitory activity among the tested nitrones. Finally, the introduction of two nitrone groups at **9a** and **9d** (bis-nitronas **9g**, **i**) did not show better neuroprotective effects than their precursor mono-nitrones. These results led us to propose nitrones containing purine (**9f**, **g**) and pyrimidine (**9a**, **c**) nucleobases as potential therapeutic agents for the treatment of cerebral ischemia and/or neurodegenerative diseases, leading us to further investigate their effects using in vivo models of these pathologies.

## 1. Introduction

**Figure 1:**
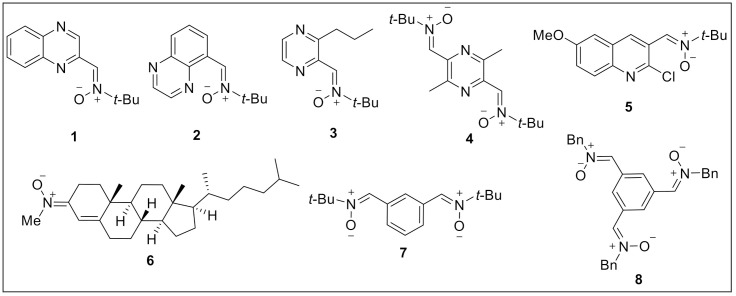
Structures of nitrones **1**–**8**.

Oxidative stress (OS) contributes to many pathological conditions, including cancer, neurological disorders [1,2,3,4], atherosclerosis, hypertension, ischemia/perfusion [5,6,7,8], diabetes, acute respiratory distress syndrome, idiopathic pulmonary fibrosis, chronic obstructive pulmonary disease [9], and asthma [10,11,12]. The OS is likely one of the most important molecular processes occurring before and after ischemic damage of membrane lipids [13]. Aerobic organisms have integrated antioxidant systems, which include enzymatic and non-enzymatic antioxidants that are usually effective in blocking the harmful effects of reactive oxygen species (ROS). However, in pathological conditions, the antioxidant systems can be overwhelmed. Consequently, current research efforts in this area are mainly focused on the identification of new, more efficient blocking and scavenging ROS [14]. In this context, free radical scavengers such as mono-nitrones **1**–**3 [15]** or bis-nitrone **4** (Figure 1) have proved to be efficient neuroprotective agents in ischemia studies [16].

**Figure 2:**
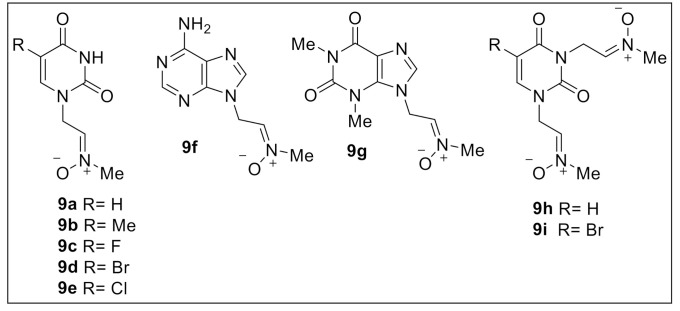
Structures of the nucleobase derived nitrones **9a**–**i** investigated in this work.

With these ideas in mind, several years ago a project was started to target the synthesis and biological evaluation of new nitrones as potential drugs for cerebral ischemia treatment [17,18]. As a result, we identified various mono-nitrones derived from (hetero)aromatic aldehydes (**5**) [19] or steroids (**6**) [20], bis-homonitrone **7** [21], and tris-homonitrone **8** [22] (Figure 1), showing high and consistent neuroprotective properties. On the other hand, some of us have studied the antiviral activity of pyrimidine-containing nitrones **9a**–**e** and the purine-containing analogues **9f**, **g** (Figure 2). Although none of the compounds were active, they appeared to be non-cytotoxic toward tested cell lines [23,24,25]. These observations inspired us to study the neuroprotective and antioxidant properties of nucleic acid base-derived mono-nitrones **9a**–**g** as well as bis-nitrones **9h**, **i** (Figure 2). As a result of this work, we have identified nitrones **9a**, **c**, **f**, **g** as the most efficient neuroprotective and antioxidant nucleobase derived nitrones. 

## 2. Results

### 2.1. Chemistry

**Scheme 1:**
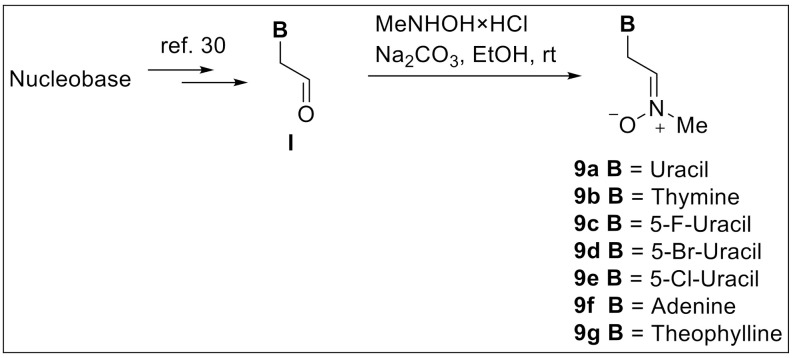
Synthesis of mono-nitrones **9a**–**g**.

**Scheme 2:**
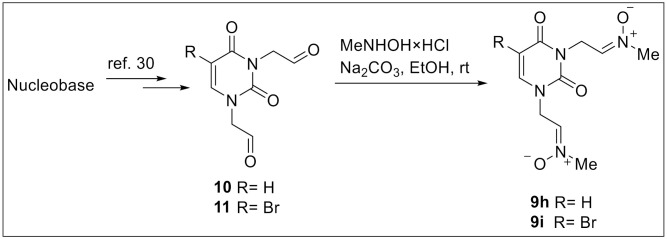
Synthesis of bis-nitrones **9h**, **i**.

Based on our previous experience with the known nitrones **9a**–**d**, **9f** [23,24], and **9g** [25] as well as new nucleobase-derived mono-nitrone **9e**, the bis-nitrones **9h**, **i** were prepared following the reactions shown in Scheme 1 and Scheme 2, respectively (Materials and Methods). Thus, the aldehydes of type **I** (Scheme 1) and the bis-aldehydes **10** and **11** (Scheme 2) were obtained directly from their respective nucleobase via mono- and dialkylation with bromoacetaldehyde diethyl acetal and subsequent acetal hydrolysis according to the literature protocol [26]. For the transformation of mono- and bis-aldehydes into the respective nitrones, **9a**–**g** and **9h**, **i**, the appropriate amounts of MeNHOH-HCl/Na_2_CO_3_ were used (2 eq. for the synthesis of **9a**–**g** and 4 eq. for **9h**, **i**). According to our previous observations, as reported earlier, all nucleobase-derived nitrones **9a**–**i** in D_2_O, DMSO-*d*_6_, and methanol-*d*_4_ solutions exist as single isomers [23]. In the NMR spectra of the corresponding nitrone **9a**–**i**, **only** signals related to the one isomer were observed (Materials and Methods). The analysis of literature data for other acyclic nitrones [27] allowed us to conclude that nitrone **9** exists as *Z*-isomers, although the corresponding signals from other *E*-isomers for comparison were never observed.

### 2.2. Neuroprotective Effects of Nitrones **9a**–**i**

#### 2.2.1. Neuroprotection Analysis in an Oxygen and Glucose Deprivation (OGD) Followed by Oxygen and Glucose Resupply (OGR) Model. Effects of Nucleobase-Derived Mono-Nitrones **9a**–**g**

Based on the number of neurodegenerative diseases where OS plays a central role, it is of paramount importance to know the effectiveness of the newly designed therapeutic agents against OS. In this study, a large-scale drug screening of nitrone compounds was performed on the SH-SY5Y cell line. OS-induced cell death in SH-SY5Y cells has been exclusively attributed to free radical overproduction [28]. 

**Figure 3:**
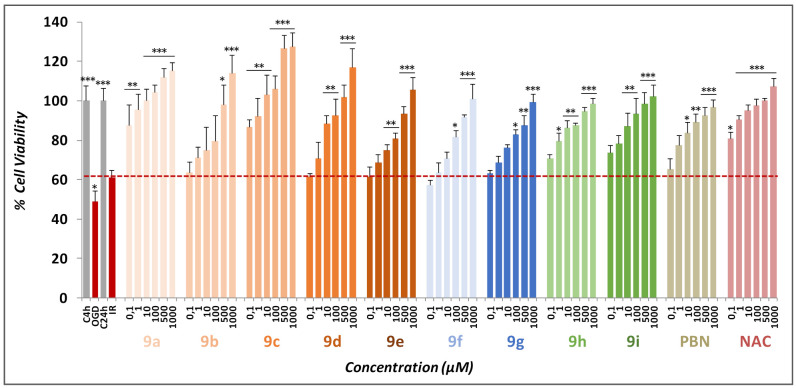
Neuroprotective effect of nitrones **9a**–**i**, α-phenyl-N-tert-butylnitrone (**PBN**), and N-acetyl-L-cysteine (**NAC**) on SH-SY5Y human neuroblastoma metabolic activity after OGD treatment (4 h) followed by OGR (24 h) (IR). Bars show percent cell viability at the indicated concentrations of nitrones **9a**–**i**, **PBN**, and **NAC**. Values represent the mean ± SEM of three experiments, each one performed in triplicate. The statistics compare the differences with IR condition alone (red dotted line) at * *p* < 0.05, ** *p* < 0.01, and *** *p* < 0.001 (one-way ANOVA, followed by Holm−Sidak analysis as a test post hoc).

The neuroprotective effect of nitrones **9a**–**g** was evaluated in an in vitro OGD model, followed by oxygen and glucose resupply (OGR) and an in vitro model of ischemia reperfusion (IR) [29]. α-phenyl-N-tert-butylnitrone (**PBN**) and N-acetyl-L-cysteine (**NAC**) were used as standards. The concentration of the tested compounds ranged from 0.01 to 1000 μM after IR. After OGD (I) (4 h), a loss of metabolic activity of about 50% (49.09 ± 5.38 % cell viability, mean ± SEM; *n* = 12) was observed, followed by a small cell recovery of about a 20–25% (62.28 ± 6.19% cell viability; mean ± SEM; *n* = 12) after 24 h reperfusion (IR) (Figure 3). Nitrones **9a**–**g** were able to partially or even completely reverse the loss of cell metabolic activity induced by OGR in a concentration-dependent manner (Figure 3). These data revealed that pyrimidinic nitrones **9a**, **c** and purinic nitrones **9f**, **g** were the most potent. Among the pyrimidinic nitrones, nitrone **9a** (with H as the R substituent) and nitrone **9c** (with F as the R substituent) (Figure 2), provided the best recovery of cell viability. However, the introduction of a methyl group in mono-nitrone **9b** or halogens (Br, Cl) in mono-nitrones **9d**, **e** decreased the neuroprotective effect. For the halogen-substituted nucleobase-derived nitrones **9d**, **e**, this reduction was proportional to the volume of the halogen substituent. The purine nitrones **9f**, **g** showed an effect on the recovery of cell viability. In summation, in the OGD experiment, mono-nitrones **9a** (B = uracil, Scheme 1), **9c** (B = 5-F-uracil, Scheme 1), **9f** (B = adenine, Scheme 1), and **9g** (B = theophylline, Scheme 1) showed the best ability to counteract the IR-induced decrease in metabolic capacity in human neuroblastoma cells. 

**Figure 4:**
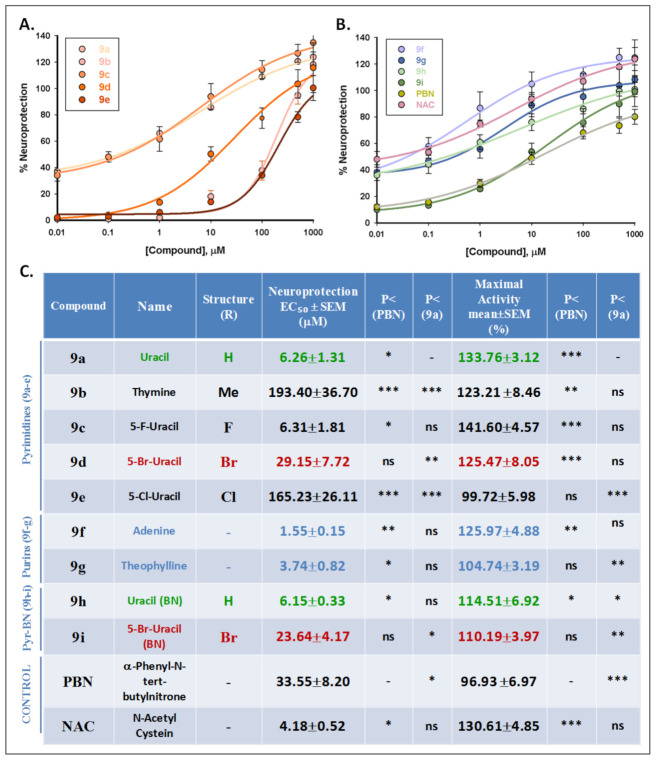
Neuroprotective effects of nitrones **9a**–**9i**, **PBN**, and **NAC** against metabolic cell damage induced by IR treatment in SHSY5Y human neuroblastoma cells. (**A**,**B**) Concentration-response curves indicating the percent of neuroprotection of different nitrones, **9a**–**e** (**A**), and nitrones **9f**–**i**, **PBN**, and **NAC** (**B**) at the specified concentrations. The curve fittings and calculations of EC_50′s_ and Maximal Activities were performed as described [21]. The data are in terms of the mean ± SEM of the four experiments, each one performed in triplicate. (**C**) EC_50_ and Maximal Activity data for the indicated compounds. Statistical comparisons of all data were carried out against **PBN** and nitrone **9a** at * *p* < 0.05, ** *p* < 0.01, and y *** *p* < 0.001 (ANOVA one way); ns—not significant.

In Figure 4 we gathered the analyses of concentration–response curves for nucleobase-derived nitrones **9a**–**g**, compared with **PBN** and **NAC**, in the range of 0.01 μM to 1 mM (Figure 4A,B), showing the corresponding EC_50_ values and the highest neuroprotective activities (Figure 4C). As shown in Figure 4C, the EC_50_ values, from lowest to highest, follows the order: **9f** ≤ **9g** ≤ **NAC** ≤ **9a** ≤ **9c** < **9d** < **PBN** ≤ **9e** ≤ **9b**, with nitrones **9f**, **9g**, **9a**, and **9d** having the highest potency, and **PBN** and nitrones **9e** and **9b** the lowest potency. There were also significant differences in the maximal neuroprotective activity of the different nitrones, from highest to lowest: **9c** **≥ 9a** **≥ 9d** **≥ NAC** > **9f** **≥ 9b** > **9e** **≥ PBN**; all nitrones had a very high maximal activity, with nitrones **9a** and **9d** having the highest potency and **9e** and **PBN** the lowest potency. 

#### 2.2.2. Effect of Nitrones **9a**–**g** on Necrotic and Apoptotic Cell Death Induced by OGD Followed by OGR Model

To further investigate the neuroprotective effect of these nucleobase-derived nitrones, we studied their neuroprotective effect on necrotic and apoptotic cell death, two types of cell death that occur during cerebral ischemia. During an ischemic stroke, there is massive cell death due to necrosis, and, consequently, the plasma membrane is broken or significantly permeabilized. Thus, necrosis can be quantified in tissue culture by measuring the release of the enzyme lactate dehydrogenase (LDH) [30]. 

**Figure 5:**
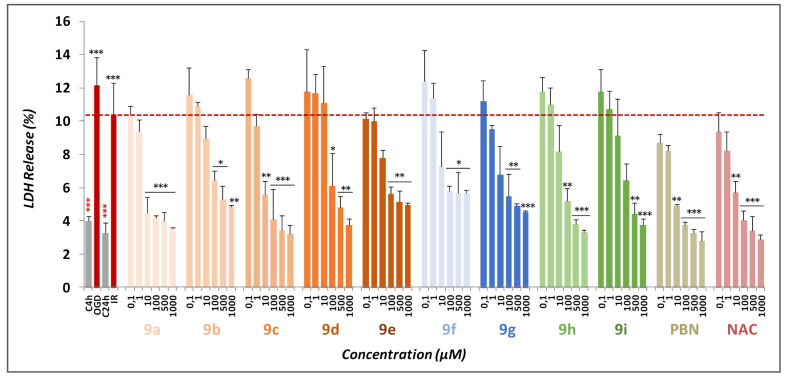
Antinecrotic effect of nitrones **9a**–**i**, **PBN**, and **NAC** in SH-SY5Y cells after OGD-OGR (IR). Bars represent cell percent LDH release after OGDR (IR) in cells treated in the absence or presence of indicated compounds and concentrations. Values represent the mean ± SEM of three experiments, carried out in triplicate, and compare the effect of OGD and IR versus their respective controls (red ***) or the effect of the different compounds after IR (24 h) with IR (24 h) alone (red dotted line) in the absence of these compounds (black ***). Statistical analysis was performed as indicated in Figure 3. * *p* < 0.05, ** *p* < 0.01, and *** *p* < 0.001.

Figure 5 shows the gathered values obtained from the measurement of LDH release after OGD for 4 h, followed by 24 h OGR (IR) on neuroblastoma cells, by adding nitrones **9a**–**i** at 0.1–1000 μM concentrations, as well as **PBN** and **NAC**. Thus, we conclude that all the nucleobase-derived nitrones significantly decreased the release of LDH in a concentration-dependent manner, reaching 100% of the maximal inhibition of LDH release at concentrations between 100 and 1000 μM (Figure 5). 

**Figure 6:**
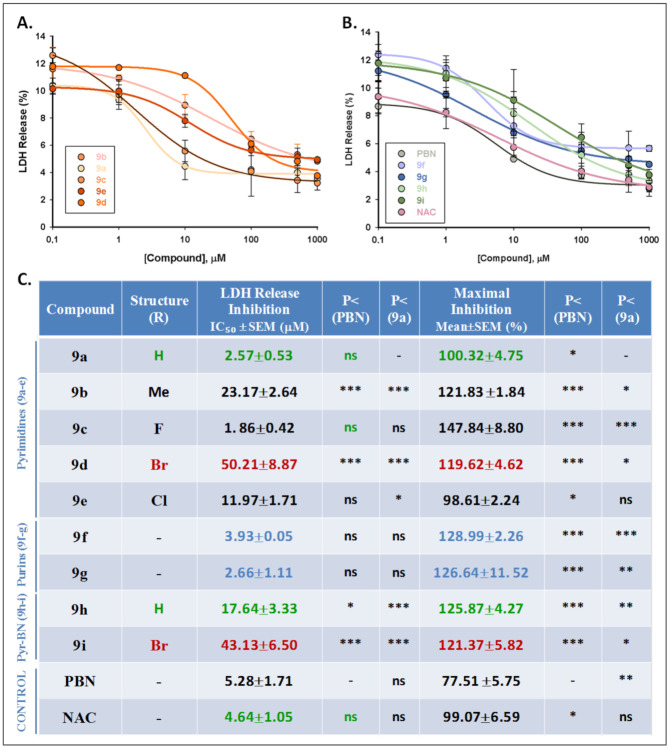
Neuroprotective effects of nitrones **9a**–**i**, **PBN**, and **NAC** against necrotic cell death induced by IR treatment in SHSY5Y cells. (**A**,**B**) Concentration-response curves showing the percent antinecrotic effect of different compounds (nitrones 9a–e (**A**) and nitrones 9f–i, **PBN** and **NAC** (**B**)) at the specified concentrations. (**C**) IC_50_ and Maximal Activity data for the indicated compounds Data representation, curve fitting, and statistical analysis of the data was performed as shown in Figure 4. Statistical differences were performed vs. **PBN**, or nitrone 9a at * *p* < 0.05, ** *p* < 0.01 y *** *p* < 0.001 (ANOVA one way); ns—not significant.

In order to compare the efficacy of these nitrones in abolishing necrotic cell death, we performed a dose-response study by determining the EC_50_ and the maximal anti-necrotic activities. Thus, Figure 6 gathers the analyses of concentration–response curves for nucleobase-derived nitrones **9a**–**g**, compared with **PBN** and **NAC**, in the range of 0.1 μM to 1 mM (Figure 6A,B), showing the corresponding EC_50_ values and the highest neuroprotective activities (Figure 6C). As shown in Figure 6C, the EC_50_ values, from lowest to highest neuroprotective nitrone follows the order: **9c** ≤ **9a** ≤ **9g** ≤ **9f** ≤ **NAC** ≤ **PBN** ≤ **9e** < **9b** < **9d**, with nitrones **9c**, **9a**, **9g**, and **9f** having the highest potency, and **PBN** and nitrones **9e**, **9b**, and **9d** the lowest potency. There were also significant differences in the maximal neuroprotective activity of the different nitrones (values from the highest to the lowest were: **9c** **≥ 9f** **≥ 9g** **≥ 9b** **≥ 9d** **≥ 9a** **≥ NAC** **≥ 9e** > **PBN**). Thus, all the nitrones had a very high maximal activity; nitrones **9c**, **9f**, and **9g** having the highest potency and nitrone **9e** and **PBN** having the lowest potency, as was the case for cell viability experiments with XTT (see below). 

Next, to evaluate the extent of cell death by apoptosis, we determined the caspase-3 activity by using DEVD-AMC as a substrate. Therefore, after OGD (4 h) and adding nitrones **9a**–**g**, **PBN**, and **NAC**, at 0.1–1000 μM concentrations, followed by IR (24 h), the cells were lysated, DEVD-AMC was added, and the fluorescence measured.

**Figure 7:**
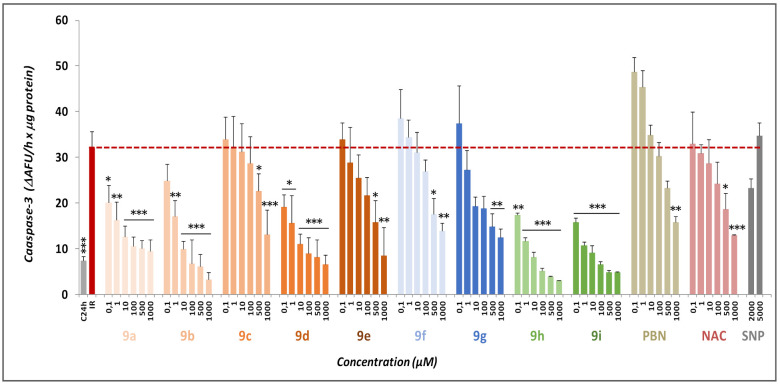
Anti-apoptotic effects of nitrones **9a**–**9i**, **PBN**, and **NAC** against IR treatment in SHSY5Y cells. Bars show caspase 3 activity (ΔAFU/min/μg protein) after OGDR (IR) treatment, in the absence or presence of **9a**–**i**, **PBN**, and NAC, at the specified concentrations. Data presentation, curve fitting, and statistical analysis of the data was performed as indicated in Figure 5. UAF = arbitrary fluorescent units. * *p* < 0.05, ** *p* < 0.01, and *** *p* < 0.001.

As shown in Figure 7, all the tested nucleobase-derived nitrones **9a**–**g** had a concentration-dependent anti-apoptotic effect. However, they generally protect less efficiently from apoptotic than necrotic cell death, and the anti-apoptotic effect profile differs from the anti-necrotic one. Nitrones with the best anti-apoptotic effect seem to be **9a** (uracil), **9b** (thymine), **9d** (Br-uracil), while those with purine nucleobases **9f**, **g** show lower anti-apoptotic than anti-necrotic power. 

#### 2.2.3. Neuroprotection Analysis of Nucleobase-Derived Bis-Nitrones **9h**, **i**

Mono-nitrone **9a** has the best anti-necrotic and anti-apoptotic properties and shows the strongest effect on neuronal metabolic activity and neuronal viability. Since we have previously seen that bis-nitrone **7** (Figure 1) has an increased neuroprotective power with respect to **PBN [23]**, we added a second nitrone motif to mono-nitrone **9a** to prepare the bis-nitrone **9h** (Scheme 2) to test its properties. For the same reason, we transformed mono-nitrone **9d**, bearing bromine at the nucleobase, into bis-nitrone **9i**. 

The analysis of the neuroprotection power of bis-nitrones **9h**, **i** has been carried out as described above for mono-nitrones **9a**–**g**. 

In the neuroprotection analysis in an OGD model followed by the OGR model, the incorporation of two nitrone motifs in the pyrimidinic mono-nitrones **9a**, **d**, with H and Br as the R substituent, respectively, leading to bis-nitrones **9h**,**i**, did not increase cell viability compared to their corresponding mono-nitrones (Figure 3). Figure 4 presents the analyses of concentration–response curves for nucleobase-derived nitrones **9h**, **i**, compared with **PBN** and **NAC**, in the range of 0.01 μM to 1 mM (Figure 4A,B), showing the corresponding EC_50_ values and the highest neuroprotective activities (Figure 4C). As shown in Figure 4C, the EC_50_ values, from the lowest to the highest, follow the order: **NAC** ≤ **9h** < **9i** ≤ **PBN**, nitrone **9h** being the most neuroprotective.

In Figure 5, we present the values obtained from the measurement of LDH release after OGD for 4 h, followed by 24 h IR on neuroblastoma cells, by adding nitrones **9h**, **i** at 0.1–1000 μM concentrations, **PBN** and **NAC**. Thus, we conclude that these nucleobase-derived nitrones significantly decreased the release of LDH in a concentration-dependent manner, reaching 100% of the maximal inhibition of LDH release at concentrations between 100 and 1000 μM (Figure 5). To compare the efficacy of these nitrones in abolishing necrotic cell death, we performed a dose-response study by determining the EC_50_ and the maximal anti-necrotic activities. Thus, Figure 6 shows the analyses of concentration-response curves for nucleobase-derived nitrones **9h**, **i** compared with **PBN** and **NAC**, in the range of 0.1 μM to 1 mM (Figure 6A,B), showing the corresponding EC_50_ values and the highest neuroprotective activities (Figure 6C). As shown in Figure 6C, the EC_50_ values, from the lowest to the highest neuroprotective nitrone, follow the order: **NAC** ≤ **PBN** < **9h** < **9i.** There were also significant differences in the maximal neuroprotective activity of the different nitrones, values which from the highest to the lowest were: **9h** **≥ 9i** > **NAC** > **PBN**. In other words, these nitrones **9h** and **9i** had the higher maximal activity. 

Next, to evaluate the extent of cell death by apoptosis, we determined the caspase-3 activity by using DEVD-AMC as a substrate, which affords fluorescent AMC upon hydrolysis. So, after OGD (4 h), and adding nitrones **9h**, **i**, **PBN**, and **NAC**, at 0.1–1000 μM concentrations, followed by IR (24 h); the cells were lysated, DEVD-AMC was added, and the fluorescence measured. As shown in Figure 7, in general, they protect less efficiently from apoptotic than necrotic cell death, and the anti-apoptotic effect profile is different from the anti-necrotic one. 

In summary, bis-nitrones **9h**, **i** are among the best nitrones, showing an anti-apoptotic effect, which improves the anti-apoptotic properties of their precursor mono-nitrones **9a** and **9d**, with H-uracil and Br-uracil as nucleobases, respectively. 

### 2.3. Basal Neurotoxicity of Nitrones ***9a**–**i***

**Figure 8:**
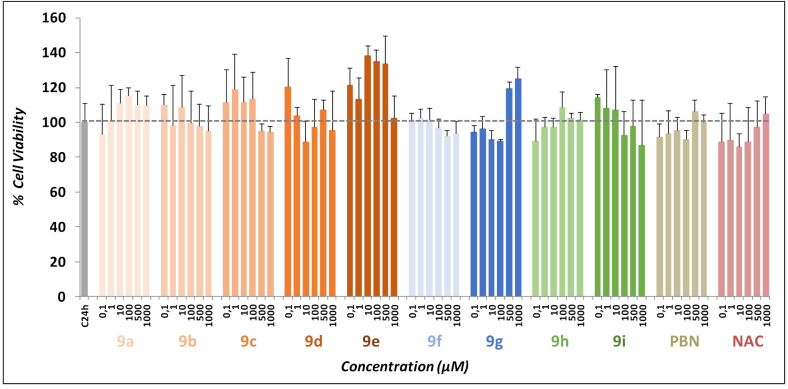
Effect of nitrones **9a**–**9i**, **PBN**, and **NAC** on human neuroblastoma SH-SY5Y cell viability under basal conditions. Bars show percent of cell viability in the presence of the compounds at the specified concentrations. The untreated cells (C24h) were considered 100% (100 ± 9.96%). Values represent the mean ± SEM of six experiments, performed in triplicate. Statistics was performed by one-way ANOVA test. There were no significant differences with respect to control. Analysis of results above 100% is not shown.

As shown in Figure 8, nitrones **9a**–**i**, as well as **PBN** and **NAC**, did not show any neurotoxic effects at basal level.

### 2.4. Antioxidant Activity of Nitrones ***9a**–**i***

#### 2.4.1. Effects of Nitrones **9a**–**i**, **PBN**, and **NAC** on Production and Scavenging of Superoxide Radical in Human Neuroblastoma SH-SY5Y Cells

O_2_^●−^ detection was carried out using dihydroethidium (DHE), after OGD (3 h) and IR (3 h), with or without nitrones **9a**–**i**, including **PBN** and **NAC** as controls. Compound concentrations from 0.1 to 1000 μM were tested after IR.

**Figure 9:**
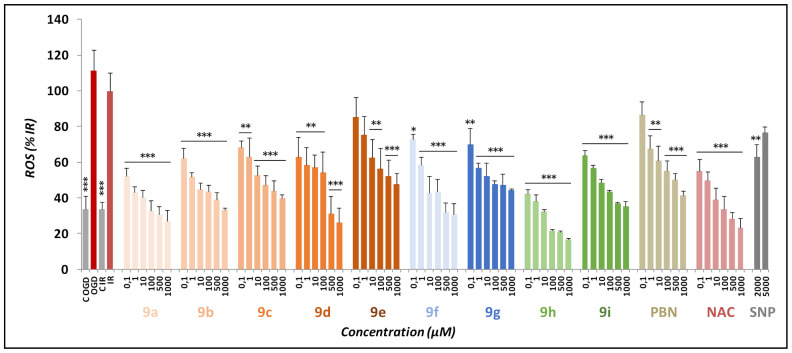
Inhibitory effects of nitrones **9a**–**i**, **PBN**, and **NAC** on superoxide production in SHSY5Y cell cultures exposed to OGD (4 h) and 3 h OGR (IR). Bars represent the percentage of ROS formed after OGD and OGR (IR), in the absence and presence of nitrones **9a**–**i** or **PBN** and **NAC**, at the specified concentrations. Values are mean ± SEM of at least four experiments, each one performed in triplicate. Data for ROS in basal conditions were of 0.101 ± 0.022 AFU/min/100,000 cells. Data presentation, curve fitting and statistical analysis of the data was performed as shown in Figure 3. The statistics compares the effect of IR versus controls or the effect of the different compounds vs. IR alone. * *p* < 0.05, ** *p* < 0.01, and *** *p* < 0.001.

As shown in Figure 9, ROS level production after IR (0.311 ± 0.057 UAF/min/100,000 cells; mean ± SEM; *n* = 12) was higher, but non-significantly different (ns, one-way ANOVA test) than ROS production under OGD alone (0.344 ± 0.065 UAF/min/100.000 cells mean ± SEM; *n* = 12). As expected, nitrones **9a**–**i** were able to partially or totally reverse the increase in ROS levels induced by IR, in a concentration-dependent manner (Figure 9). 

**Figure 10:**
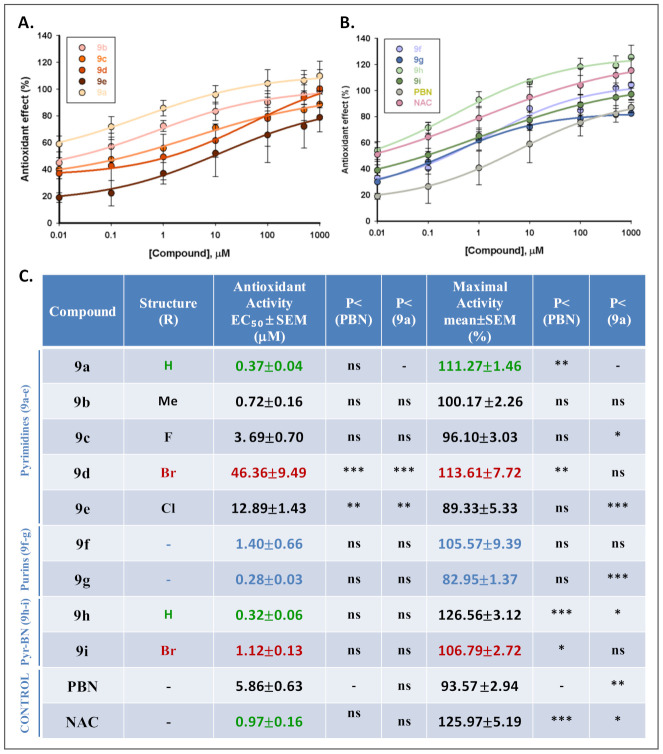
Antioxidant effect of nitrones **9a**–**i**, **PBN** and **NAC** after OGDR (IR) in human neuroblastoma SH-SY5Y cells. (**A**,**B**) Dose–response curves showing the percentage of antioxidant effect of different compounds at the specified concentrations. (**C**) EC_50_ values and maximal antioxidant activities for the indicated compounds. Data representation, curve fitting, and statistical analysis of the data was performed as shown in Figure 4. The statistics compare the differences between EC_50_ or maximal activities values for different compounds studied versus **PBN** or nitrone **9a**. * *p* < 0.05, ** *p* < 0.01, and *** *p* < 0.001; ns—not significant.

The analyses of the concentration-response curve data (Figure 10A,B) and calculations of EC_50_ and the highest antioxidant activities of nitrones **9a**–**i**, **PBN**, and **NAC** are shown in Figure 10C. The EC_50_ values, from lowest to highest, follow the order: **9g** ≤ **9h** ≤ **9a** ≤ **9b** ≤ **NAC** ≤ **9i** ≤ **9f** ≤ **9c** ≤ **PBN** < **9e** < **9d**, with nitrones **9g**, **9h**, and **9a** having the highest potency and **PBN** and nitrones **9e** and **9d** having the lowest potency. There were also significant differences in the maximal neuroprotective activity of the different nitrones, with values from highest to lowest as follows: **9h** ≥ **9a** ≥ **NAC** ≥ **9d** ≥ **9a** ≥ **9f** ≥ **9c** ≥ **PBN** ≥ **9e**. In other words, all the nitrones had a very high maximal activity, with nitrones **9h** and **9a** having the highest potency and **PBN** and nitrone **9e** having the lowest potency, as was the case for cell viability experiments with XTT. However, despite these small differences in the EC_50_ values of the antioxidant effect, the application of statistical tests for comparison shows that only nitrones **9d**, **e**, with Br and Cl substituted uracil as nucleobase, respectively, were significantly higher than **PBN** and nitrone **9a**. As for the maximal antioxidant capacity, only nitrones **9a**, **d**, **h**, **I**, and **NAC** were significantly higher than **PBN** and only nitrones **9c**, **e**, **g**, and **PBN**, significantly lower than nitrone **9a**, being those of **NAC** and nitrone **9h** significantly higher than this nitrone. 

In summary, we can conclude that once again the mono-nitrones **9a**–**c** bearing the pyrimidine nucleobase and **9f**, **g** bearing the purine nucleobase have similar antioxidant capacity also very similar to that of NAC. However, once again, the substitution of the H at C5 of the uracil motif by Br or Cl from mono-nitrone **9a** to nitrones **9d**, **e** lowers their antioxidant power. The addition of a second nitrone group to the mono-nitrones **9a**, **d**, with H and Br substituent, as radical, leading to nitrones **9h**, **i**, respectively, does not modify the antioxidant capacity of nitrone **9a** with uracil as nucleobase. However, it seems to significantly improve the antioxidant capacity of nitrone **9d**, with Br-uracil as nucleobase, although only in respect to the EC**_50_**. 

In conclusion, the pyrimidinic mono-nitrones **9a** and **9c**, and, in this case **9b**, and the purine mono-nitrones **9f** and **9g**, are the nitrones that present the best antioxidant properties. This conclusion is consistent with their neuroprotective properties on neuronal metabolic and antinecrotic capacity but not with its anti-apoptotic activity. Regarding the addition of new nitrone groups to these nucleobase-substituted nitrones, globally, it seems to provide, and only in the case of nitrone **9i** over **9d**, an increase in its antioxidant capacity, although this increased antioxidant capacity is not able of improving its neuroprotective effect. 

Next, we analyzed the inhibition of LOX, the inhibition of lipid peroxydation (ILPO), the trapping of DPPH radical, the scavenging of hydroxyl free radicals, and the decolorizing ABTS test by nitrones **9a**–**i** and the appropriate standards.

#### 2.4.2. Effects of Nitrones **9a**–**i**, **PBN** and **NAC** in the Inhibition of LOX, ILPO, Scavenging DPPH, Hydroxyl Radicals, and the Decolorizing ABTS Test

5-LOX is a crucial enzyme in the arachidonic cascade that leads to the production of leukotrienes [31], important inflammation mediators. Therefore, they are involved in the pathobiology of stroke injury. LOXs induce the lipid peroxidation of membranes producing hydroperoxides [32]. Lipid peroxidation is propagated by cerebral ischemia-reperfusion and inflammation. LOX inhibition is gaining interest as a molecular target for the treatment of inflammatory diseases and cardiovascular pathologies [32]. In vitro LOX inhibition was performed by the UV absorbance-based enzyme assay. Considering the IC_50_ inhibition values in Table 1, it seems that mono-nitrones **9c** (28 μΜ), **9f** (31 μΜ), and **9g** (33 μΜ), are the most active derivatives, quite far from the value shown by standard **NDGA** (0.45 μΜ). However, bis-nitrone **9h** was inactive and **9i** showed lower IC_50_ value (57.5 μΜ). Although lipophilicity is described as a significant physicochemical parameter for LOX inhibition [24], within this series we did not find any positive correlation with lipophilicity. Within this series the calculated clog *p* values point to their hydrophilic character (**9a** > **9f** > **9c** > **9b** > **9g** > **9e** > **9d >> 9h** > **9i**). Thus, **9i** is more of a lipophilic compound than **9a**, which is the most hydrophilic/less lipophilic. However, **9i** does not present the higher LOX inhibitory activity. On the contrary, **9c**, a less lipophilic compound, shows higher LOX inhibition. 

Antioxidant capacity assays can be classified into two types based on HAT (hydrogen atom transfer) reactions and ET (electron transfer). HAT-based assays include the AAPH assay, a well-documented method applied in vitro for radical-scavenging activity measurements. The main reason for this is that the peroxyl radicals formed by the reaction of AAPH resemble to the cellular lipid peroxidation [33]. Lipid peroxidation propagates the ROS-mediated injury, which is directly harmful to membranes. In the AAPH assay, the highly reactive alkylperoxyl radicals are intercepted by a HAT from the antioxidant. Therefore, particularly effective HAT agents are compounds with high hydrogen atom donating ability, that is, compounds with low heteroatom-H bond dissociation energies and/or compounds from which hydrogen abstraction leads to sterically hindered radicals, as well as compounds from which abstraction of hydrogen leads to C-centered radicals stabilized by resonance. Thus, we examined the anti-lipid peroxidation behaviour of nitrones **9a**–**g** using the AAPH protocol. In our experiments, compounds **9g**, **9f**, and **9d** significantly inhibited lipid peroxidation (ILPO = 92–84%) at 100 μM (Table 1). Trolox was used as a positive control (93%). Bis-nitrone **9h** was inactive. Bis-nitrone **9i** significantly inhibited the lipid peroxidation, showing a value (71%) lower than the observed (84%) for the corresponding mono-nitrone **9d**. No role was found for lipophilicity. 

**PBN** was inserted with its results in Table 1 as a reference compound and for comparison reasons.

The interaction of nitrones with DPPH was studied in two different concentrations (50 μM and 100 μM) and time periods (20 and 60 min). No result was observed at 50 µM, whereas a very low result was recorded at 100 µM for mono-nitrones **9a**–**g** (6–20%, Table 1). However, bis-nitrones **9h** (82%) and **9i** (76%) (Table 1) present high reducing ability compared to the corresponding mono-nitrones **9a** (20%) and **9d** (6%) (Table 1). This increase is possibly related to the presence of the two nitrone motifs. Higher reducing activities were indicated by the two more lipophilic compounds, **9h** and **9i**.

The hydroxyl (^•^OH) free radical is characterized as the most toxic among the ROS. As shown in Table 1, the results underline that the majority of the tested nitrones presented significantly high activity at 100 μM, in some cases with scavenging activities higher (87–99%) than Trolox (73%), the reference compound. Considering the subgroup of nucleobase-derived nitrones **9a**–**g**, nitrone **9c** (99%) is most potent, followed by **9d** (90%) and **9e** (87%), whereas mono-nitrones **9f** and **9g** showed significant lower trapping values. The absence of a substituent at C-5 in the pyrimidine moiety of the nitrones **9a**–**e** vanishes the scavenging activity, e.g., **9a** (no activity), while the size of 5-substituent influences activity. Smaller substituents like F **(9c**) lead to higher competition (99%), whereas Cl (**9e**) or CH_3_ (**9b**) present lower activity, whereas bis-nitrone **9h** showed limited activity (25%), and **9i** was inactive (Table 1). No role for lipophilicity was found.

The ABTS assay is another method used to determine the antioxidant activities based on the free radical scavenging. The oxidation of ABTS by potassium persulfate [34] gives rise to the derivation of the ABTS cationic radical. The presence of electron-donating antioxidants in a solution leads to its reduction. Trolox has been used as a reference compound. Mono-nitrones **9a**–**g** presented limited activity (Table 1). On the contrary, bis-nitrones **9h** (50%) and **9i** (75%) (Table 1) are significantly potent due to the presence of the two nitrone groups. The presence of bromine enhances activity. Higher antioxidant activities were indicated by the two more lipophilic compounds, **9h** and **9i**.

## 3. Discussion and Conclusions

In this work we have reported the synthesis and neuroprotection profile of nucleobase-derived nitrones **9a**–**i**. 

From the results of the neuroprotection experiment under OGD conditions followed by the OGR model (Figure 3 and Figure 4), and in terms of structure-activity relationship (SAR), we conclude that: (1) The nitrones with the highest neuroprotective capacity are those containing a purine nucleobase (nitrones **9f**, **g**), and among the pyrimidine nucleobase nitrones, nitrone **9a**, the pyrimidine base with no substituent at C5 position and nitrone **9c** (with F as the R substituent). This neuroprotective capacity is very similar to that of **NAC**, both in terms of EC_50_ (in the range of 1 μM) and maximal neuroprotective activity (greater of 100%), and much higher than that of the control nitrone **PBN** (EC_50_ ≈ 35 μM and maximal activity of ≈ 96%). (2) The neuroprotective capacity of these nitrones (EC_50_) is worsened by substituting the pyrimidine nucleobases. Thus, the replacement of H at C5 of the uracil by a methyl group (thymine) produces the greatest decrease in activity (with EC_50_ increasing from 2 μM to about 200 μM). On the other hand, the introduction of halogen substituents at C5 in the uracil structure also worsens the neuroprotective activity of the pyrimidine nucleobase-derived nitrone, a worsening that seems proportional to the size of the halogen. In other words, there is no significant variation in neuroprotective activity when F is incorporated (nitrone **9c**). However, this capacity decreases when Br and Cl are incorporated (nitrones **9d**, **e**), and the EC_50_ increases from 1 μM to 35 μM and 170 μM, respectively, although these nitrones retain the same maximal activity as the nitrone **9a** having unsubstituted uracil. (3) The incorporation of a second nitrone function into the structure of uracil nitrone **9a** and 5-Br-uracil nitrone **9d** gives rise to two bis-nitrones, **9h**, **i** respectively, which do not significantly improve the neuroprotective characteristics of their respective precursor nitrones, neither in EC_50′_s nor in maximal activities. (4) Finally, the nucleobase-derived nitrones **9a**-**i** have better neuroprotective activities than **PBN** (especially **9a**, **f**, **g**) and are very similar to those of the well-known neuroprotective agent **NAC.**

Based on the observed efficacy of nitrones **9a**–**i** in abolishing necrotic cell death (Figure 5 and Figure 6) and from the SAR point of view, we found the following differences when comparing the results of metabolic capacity of these nucleobase-derived nitrones (cell viability, measured with XTT): (1) As observed in the metabolic capacity results, the nitrones with the highest neuroprotective capacity (EC_50_) are those containing a purine nucleobase (nitrones **9f**, **g**) and those containing a pyrimidine base and no substituents at the C5 position (nitrone **9a**). However, nitrone **9c**, with 5-F-uracil as the nucleobase, improves the characteristics of the mono-nitrone **9a**, thus, becoming one of the four best mono-nitrones studied with an EC_50_ in the range of 1 μM. Mono-nitrone **9c** also shows the highest maximum activity, in the same range that purine-nitrones **9f**, **g**, but not nitrone **9a**, showed a lower capacity. This anti-necrotic capacity is better and very similar to that of **NAC**, which showed a close EC_50_ but lowered maximal activity than in terms of recovery of cellular metabolic activity. Moreover, this anti-necrotic capacity is also close to **PBN**, showing an EC_50_ in the same range, although it was the worst in terms of its maximal anti-necrotic activity (EC_50_ ≈ 5 μM and Maximal Activity of ≈ 75%). (2) As observed in the metabolic capacity results, among the nitrones derived from pyrimidine nucleobase, the anti-necrotic capacity (EC_50_) is worsened by the addition of radicals at the R-position. Thus, the replacement of H at C5 in uracil by a Me group (thymine) produces the greatest decrease in activity (with EC_50_ increasing from 2 μM to about 25 μM; one order of magnitude). On the other hand, the incorporation of halogens, such as Br (**9d**) and Cl (**9e**), in the uracil motif also worsens the anti-necrotic activity of nitrones with pyrimidine-nucleobase. This worsening is greater in the case of substitution by Br instead Cl, but not observed for the nucleobase-derived nitrone bearing a F group (**9c**). This effect only seems to affect the EC_50_, but not the maximal anti-necrotic activity, when compared to nitrone **9a**, although it is significantly lower when compared to the maximal activity of the best F-substituted pyrimidine nitrone (**9c**). (3) The introduction of a second nitrone group into the structure of uracil and 5-Br-uracil (bis-nitrones, **9h**, **i** respectively) does not improve the anti-necrotic properties of its precursors, neither in terms of EC_50_, nor in terms of maximum anti-necrotic activity, but rather seems to worsen it in the case of nitrone **9h**. (4) In summary, nucleobase-derived nitrones **9a**, **c**, **f**, **g** have better anti-necrotic power than **NAC** and **PBN**.

The results of the anti-necrotic and anti-apoptotic power of the investigated nitrones are interesting and can be summarized as follows: (1) The anti-necrotic and anti-apoptotic properties of the different nitrones substituted with nucleobases influence the general metabolic state of the cell in different ways: those with the best anti-necrotic properties (uracil pyrimidine mono-nitrone **9a** and the purine mono-nitrones **9h**, **i**), most favorably influence the general cellular metabolic state, while the anti-apoptotic effect influences it less. (2) Having two nitrone groups in the same compound, despite the improvement of the anti-apoptotic effect of their precursor nitrones, would not be sufficient to recover the general cellular metabolic state and, therefore, not sufficient to improve the neuroprotective properties of their precursor mono-nitrones. (3) Mono-nitrone **9a**, which has the best anti-necrotic and anti-apoptotic properties, is the one that shows the best neuronal metabolic activity and neuronal viability.

The antioxidant activities of nucleobase-derived nitrones **9a**–**i** have been analyzed by determining their effects on the production and scavenging of superoxide radical in human neuroblastoma SH-SY5Y cells. Once again, the pyrimidinic mono-nitrones **9a** and **9c**, and, in this case also **9b**, and the purine mono-nitrones **9f** and **9g** show the best antioxidant properties, which is consistent with their neuroprotective properties on neuronal metabolic capacity and with its antinecrotic capacity, and not with its anti-apoptotic activity. 

The antioxidant activities of nucleobase-derived nitrones **9a**–**i** have also been investigated by using different antioxidant assays, such as the inhibition LOX, the inhibition of lipid peroxidation induced by the AAPH, the interaction with the free, stable radical DPPH, their competition with DMSO for hydroxyl radicals, the scavenging of the cationic radical ABTS in comparison to **PBN**, and the scavenging of the cationic radical ABTS. Most of these nitrones highly compete with DMSO for hydroxyl radicals, considering them as strong quenching agents, and exhibit in vitro anti-lipid peroxidation activities. Nitrones **9c**, **9f**, and **9g** combine the higher lipoxygenase IC_50_ values within the presented group. A favorable antioxidant profile can be considered a convenient agent for the development of functional bioactive molecules for the treatment of AD or stroke, aging diseases correlated to ROS, and neuroinflammation.

In conclusion, the combination of purine and pyrimidine nucleotide bases with a moiety of one or two nitrone groups gives rise to new nucleobases-containing nitrones with better antioxidant and neuroprotective capacities than nitrones, such as **PBN** or HomoBisNitrones derived of PBN (**HBNs**), alone. This could be due to the combination of the antioxidant properties of nitrones with the activation of the synthesis of uracil and adenine nucleotides, which, when phosphorylated inside the cell, enhance the energetic and metabolic cell capacity (by synthesis of high-energy molecules such as ATP or sugar-transporters like UTP or the synthesis of nucleic acids by enhancing activity of enzymes as the ribonucleotide-reductase) [35]. We have discovered pyrimidinic nitrones **9a** and **9c**, and purinic nitrones **9f** and **9g**, as nitrones with better antioxidant and neuroprotective properties than **PBN**, and even that **HBNs**, previously tested in our research group [21], that could be good candidates to be proposed as potential molecules for stroke therapeutics. It has been reported that the combination of a tetramethylpyrazine derivative with a potent ROS-scavenger as nitrone scaffold diminishes the size of cerebral infarction and the neurological deficit in rats and non-human primate models of stroke through multi-factorial modes of action [36]. Therefore, further investigation on the activity of these nucleobase-derived nitrones in in vivo models of cerebral ischaemia would be highly desirable.

## 4. Materials and Methods

### 4.1. Chemistry

#### 4.1.1. General Procedure for the Preparation of Nitrones **9a**–**g**

The corresponding aldehyde (1.00 mmol) was dissolved in ethanol (5 mL) and then an aqueous solution of methylhydroxylamine hydrochloride (2 mmol) (Sigma-Aldrich, Poznan, Poland) was added, followed by sodium carbonate (1.5 mmol) (Sigma-Aldrich, Poznan, Poland). The reaction mixture was kept at room temperature (r.t.) for 4 h. After removal of solvents, the crude product was purified by column chromatography (CHCl_3_-MeOH, 10:1, *v*/*v*) to obtain the respective pure nitrone **9a**–**g [23,24,25].**

#### 4.1.2. Compound **9b** (B = Thy)

The compound was prepared from 1-(2-oxoethyl)thymine. Amorphous solid; mp 182 °C (decomp.); IR (KBr) ν_max_ 3441, 3109, 3006, 2816, 2615, 1677, 1470, 1418, 1310, 1225, 992 cm-1; ^1^H NMR (600 MHz, D2O) δ 7.51 (s, 1H, HC6), 7.41 (t, 1H, *J* = 4.4 Hz, CH–CH_2_), 4.66 (d, 2H, *J* = 4.4 Hz, CH_2_), 3.72 (s, 3H, CH_3_), 1.87 (3, 3H, CH_3_); ^13^C NMR (151 MHz, D_2_O) δ 166.98, 152.25, 142.99, 141.53, 111.01, 51.43, 45.58, 11.36. Anal. Calcd. for C_8_H_11_N_3_O_3_×0.25H_2_O: C, 47.64; H, 5.75; N, 20.83. Found: C, 47.83; H, 5.69; N, 20.83.

#### 4.1.3. Compound **9c** (B = F-Ura)

The compound was prepared from 1-(2-oxoethyl)-5-fluorouracil. Amorphous solid; mp 195 °C (decomp.); IR (KBr) ν_max_ 3385, 3119, 3053, 3022, 2958, 2714, 2577, 1695, 1613, 1453, 1374, 1249, 1007 cm-1; ^1^H NMR (300 MHz, D2O) δ 7.90 (d, 1H, *J* = 5.7 Hz, HC6), 7.45 (t, 1H, *J* = 4.8 Hz, CH–CH_2_), 4.65 (d, 2H, *J* = 4.8 Hz, CH_2_), 3.72 (s, 3H, CH_3_); ^13^C NMR (151 MHz, D_2_O) δ 160.08 (d, *J* = 25.4 Hz), 150.92, 140.97, 140.46 (d, *J* = 232.7 Hz), 131.28 (d, *J* = 34.0 Hz), 51.46, 45.94. Anal. Calcd. for C_7_H_8_FN_3_O_3_: C, 41.80; H, 4.01; N, 20.89. Found: C, 41.80; H, 3.81; N, 21.00.

#### 4.1.4. Compound **9d** (B = Br-Ura)

The compound was prepared from 1-(2-oxoethyl)-5-bromouracil. Amorphous solid; mp 180 °C (decomp.); IR (KBr) ν_max_ 3069, 3020, 2933, 2657, 1705, 1678, 1620, 1431, 1330, 1201, 969 cm-1; ^1^H NMR (600 MHz, DMSO-*d*_6_) δ 8.22 (s, 1H, HC6), 7.26 (t, 1H, *J* = 4.7 Hz, CH–CH_2_), 4.48 (d, 2H, *J* = 4.7 Hz, CH_2_), 3.60 (s, 3H, CH_3_); ^13^C NMR (151 MHz, DMSO-*d*_6_) δ 160.24, 150.82, 146.23, 134.86, 94.85, 52.21, 45.50. Anal. Calcd. for C_7_H_8_BrN_3_O_3_: C, 32.08; H, 3.08; N, 16.03. Found: C, 32.39; H, 3.09; N, 16.33.

#### 4.1.5. Compound **9e** (B = Cl-Ura)

The compound was prepared from 1-(2-oxoethyl)-5-chlorouracil. Amorphous solid; mp 180 °C (decomp.); IR (KBr) ν_max_ 3424, 3021, 2958, 2925, 2852, 1683, 1625, 1430, 1265 cm-1; ^1^H NMR (600 MHz, D2O) δ 8.03 (s, 1H, HC6), 7.44 (t, 1H, *J* = 4.8 Hz, CH–CH_2_), 4.71 (d, 2H, *J* = 4.8 Hz, CH_2_), 3.74 (s, 3H, CH_3_); ^13^C NMR (151 MHz, D_2_O) δ 162.42, 151.47, 144.62, 140.71, 108.23, 51.49, 45.97. Anal. Calcd. for C_7_H_8_ClN_3_O_3_: C, 38.64; H, 3.71; N, 19.31. Found: C, 38.56; H, 3.41; N, 19.36.

#### 4.1.6. Compound **9f** (B = Ade)

The compound was prepared from 1-(2-oxoethyl)adenine. Amorphous solid; mp 211 °C (decomp.); IR (KBr) ν_max_ 3352, 3271, 3243, 3055, 2921, 2748, 2687, 1678, 1601, 1579, 1480, 1300 cm-1; ^1^H NMR (600 MHz, D2O) δ 8.18 (s, 1H, HC2), 8.15 (s, 1H, HC8), 7.54 (t, 1H, *J* = 4.8 Hz, CH–CH_2_), 5.19 (d, 2H, *J* = 4.8 Hz, CH_2_), 3.75 (s, 3H, CH_3_); ^13^C NMR (151 MHz, D_2_O) δ 155.43, 152.65, 148.85, 142.36, 140.56, 118.11, 51.45, 40.43. Anal. Calcd. for C_8_H_10_N_6_O×0.15H_2_O: C, 45.99; H, 4.97; N, 40.23. Found: C, 46.16; H, 4.74; N, 40.28.

#### 4.1.7. Compound **9g** (B = Theophylline)

The compound was prepared from 9-(2-oxoethyl)theophylline. Amorphous solid; mp 192 °C (decomp.); IR (KBr) ν_max_ 3350, 3274, 3243, 3090, 2941, 2677, 1668, 1601, 1570, 1481, 1290 cm–1; ^1^H NMR (300 MHz, D_2_O) δ 8.05 (s, 1H, HC8), 7.53 (t, 1H, *J* = 4.4 Hz, CH–CH_2_), 5.26 (d, 2H, *J* = 4.4 Hz, CH_2_), 3.72 (s, 3H, CH_3_), 3.51 (s, 3H, CH_3_), 3.31 (s, 3H, CH_3_); ^13^C NMR (75.5 MHz, D_2_O) δ 155.72, 152.43, 148.56, 143.89, 140.55, 107.09, 51.44, 43.42, 29.94, 27.96. Anal. Calcd. for C_10_H_13_N_5_O_3_: C, 47.81; H, 5.22; N, 27.88. Found: C, 47.58; H, 5.13; N, 27.59.

#### 4.1.8. General Procedure for the Preparation of Nitrones **9h**,**i**

The corresponding aldehyde 10 and 11 (1.00 mmol) were dissolved in ethanol (5 mL) and then an aqueous solution of methylhydroxylamine hydrochloride (4 mmol) was added, followed by sodium carbonate (3 mmol). The reaction mixture was kept at room temperature (r.t.) for 4 h. After removal of solvents, the crude product was purified by column chromatography (CHCl_3_-MeOH, 10:1, *v*/*v*) to obtain the respective pure nitrone 9h, i.

#### 4.1.9. Nitrone **9h** (B = Ura)

Prepared from 1,3-di(2-oxoethyl)uracil. Amorphous solid; mp 190 °C (decomp.); IR (KBr) ν_max_ 3395, 3096, 2956, 2924, 2853, 1705, 1658, 1452, 1399, 1242 cm^−1^; ^1^H NMR (200 MHz, CD_3_OD) δ 7.76 (d, *J* = 7.9 Hz, 1H, CH = CH), 7.42–7.37 (m, 1H, CHN), 7.25–7.19 (m, 1H, CHN), 5.82 (d, *J* = 7.9 Hz, 1H, CH = CH), 4.91–4.87 (m, 2H, CH_2_), 4.71–4.66 (m, 2H, CH_2_), 3.76 (d, *J* = 0.9 Hz, 3H, CH_3_), 3.73 (d, *J* = 0.9 Hz, 3H, CH_3_); ^13^C NMR (151 MHz, CD_3_OD) δ 163.43, 151.48, 144.87, 140.26, 137.54, 100.05, 50.86, 50.47, 38.01. 

#### 4.1.10. Nitrone **9i** (B = Br-Ura)

Prepared from 5-bromo-1,3-di(2-oxoethyl)uracil. Amorphous solid; mp 198 °C (decomp.); IR (KBr) ν_max_ 3418, 3095, 2957, 2924, 2854, 1712, 1658, 1443, 1175, 760 cm^−1^; ^1^H NMR (600 MHz, CD_3_OD) δ 8.23 (s, 1H, CH = CBr), 7.36–7.33 (m, 1H, CHN), 7.20–7.17 (m, 1H, CHN), 4.92–4.90 (m, 2H, CH_2_), 4.70–4.68 (m, 2H, CH_2_), 3.74 (d, *J* = 0.7 Hz, 3H, CH_3_), 3.72 (d, *J* = 0.7 Hz, 3H, CH_3_); ^13^C NMR (151 MHz, CD_3_OD) δ 159.56, 150.78, 144.57, 139.68, 137.07, 94.11, 50.90, 50.54, 39.25. 

### 4.2. Neuroprotection Assays

#### 4.2.1. Neuroblastoma Cell Cultures

Cultures of human neuroblastoma cells from line SH-SY5Y were performed in flasks containing Dulbecco’s: Ham’s F12, 1:1 [*v/v*] composed of 0.5 mM sodium pyruvate, 2.5 mM GlutaMAX™; GIBCO, Life Technologies, Madrid (Spain), 3.15 mg/mL glucose, 1% Antibiotic-Antimycotic (100 ui/mL penicillin, 100 mg/mL streptomycin, and 0.25 mg amphotericin B), 1% Gentamicin 15 mg/mL (Sigma-Aldrich, Madrid, Spain) and 10% Fetal Calf Serum (FCS) (Gibco; Life Technologies, Madrid, Spain) as as described [21]. For each experiment, neuroblastoma cells were digested partially with 0.25% trypsin-EDTA and seeded in plates of 96 or 48 wells with a density of 0.5–1 or 2–2.5 × 10^5^ cells/well, respectively, depending on the assay. At 80% confluence of the seeded cells, medium was renewed adding 0.1–1000 µM of the compounds tested.

#### 4.2.2. Neuroblastoma Cell Cultures Exposure to Oxygen–Glucose Deprivation (OGD)

To induce experimental ischemia, and the sub-consequent cellular damage, the neuroblastoma cultures underwent OGD for 3–4 h, maintained in a glucose-free Dulbecco’s medium inside an anaerobic chamber in the presence of a gas mixture of 95% N_2_/5% CO_2_ and humidified at 37 °C at a constant pressure of 0.15 bar. After the OGD period (4 h), medium was substituted by oxygenated 10% FCS-containing medium, and cells were incubated in conditions of normoxia for 24 h to recovery (R24h). The compounds used in the neuroprotection studies: nitrones **9a**–**i**, α-phenyl-N-tert-butyl nitrone (**PBN**) and N-acetyl-L-cysteine (**NAC**) (0.01 μM−1000 µM), were added at the beginning of R24h as described [21]. Concurrently, controls in the absence of the OGD procedure were performed by maintaining these cells with Dulbecco’s medium including glucose for 3–4 h in an incubator in conditions of normoxia during the OGD period; thereafter, the medium was changed, and the cells (in the presence or absence of the different study compounds) were returned to the incubator for 24 h of recovery, as described [21]. In addition, experiments including the same amounts of vehicle (final concentration < 1% dimethyl sulfoxide) were performed [21], serving as controls. The effects of nitrones **9a**–**g** were analyzed three-five times independently using different culture batches, and each experiment was carried out in triplicate.

#### 4.2.3. Evaluation of Cell Viability

Cell viability measurements were carried out in human neuroblastoma cells SHSY5Y using 96-well culture plates, containing about 0.5–1 × 10^5^ cells/well, after the OGD-R24h or control treatment including 0.01–1000 µM of the different testing compounds (nitrones **9a**–**g**, **PBN**, and **NAC**), utilizing the Cell Proliferation Kit II (XTT), Sigma Aldrich, Madrid, Spain, which determines the culture’s metabolic capacity, as described in [21,22]. XTT solution (0.3 mg/mL final concentration) was added to the seeded cells, incubated for 2 h at 37 °C with 5% CO_2_ and 95% air (*v*/*v*), and a spectrophotometer reader (Biotek Power-Wave XS microplate-reader (BioTek Instruments, Madrid, Spain) was used to register the absorbance at 450 nm (reference 650 nm) of the soluble formazan product generated. 100% viability was set by control normoxic cells treated with medium only, and control cells were added 0.001–1% DMSO in every assay.

#### 4.2.4. Assessment of LDH Activity

Anti-necrotic cell activity was evaluated by culturing SHSY5Y cells in 48-well dishes, in a density of 2 × 10^5^ cells/well and treating them as controls or under the OGD-R24h procedure, adding the testing compounds at 0.01–1000 µM (see point 4.2.2.). Media of every well were collected in Eppendorf tubes properly labelled and stored at −20 °C until measurement (samples would be needed to measure extracellular NADH). Afterwards, each well was recharged with a 0.1 M Phosphate Buffer with 0.5% Triton X-100, pH 7.5, in order to scratch the cells from the bottom of the well. After a 13.000 rpm centrifugation, the soluble fractions are collected and labelled (samples would be essential to measure intracellular NADH). Finally, LDH activity was quantified as the degree of absorbance declined at 340, indicating the oxidation of NADH to NAD+ as described [21,22]. Results are expressed as the LDH activity of the extracellular medium referred to the total LDH activity after cell lysis.

#### 4.2.5. Measurement of Caspase-3 Activity

For apoptosis assays, human neuroblastoma cells were seeded in 48-well culture plates (density 2–2.5 × 10^5^ cells/well) and underwent the OGD procedure or were treated as controls (described previously). After the treatment, cells were incubated in the presence of the different nitrones and compounds at 1–500 µM or used as positive controls of damage for 24 h (R24h). Afterwards, cells were lysed at 4 °C in a lysis medium containing 5 mM Tris/HCl (pH 8.0), 20 mM ethylenediaminetetraacetic acid, and 0.5% Triton X-100, and centrifuged at 13.000 rpm for 10 min. Specific Caspase-3 activity expressed as arbitrary fluorescence units ((AFU)/µg protein/h), was assessed by making use of a fluorigenic substrate peptide DEVD-AMC (66081; BD Biosciences PharMingen, Madrid, Spain), and by considering protein quantity determined using the Bradford method, as described previously [21].

#### 4.2.6. Evaluation of ROS Formation

A density of 2–2.5 × 10^5^ SHSY5Y cells/well was used to seed in 48-well plates to quantify superioxide anion formation. Firstly, cultured cells were exposed to OGD treatment for 3–4 h or maintained in normoxic conditions (controls). Thereafter, medium was replaced with oxygenated Dulbecco’s: Ham’s F12 medium with glucose, 1% Antibiotic-Antimycotic, 1% Gentamicin and 10% FCS. Cells were incubated in the absence (controls) or presence of indicated concentrations of the compounds of study, at 37 °C for 3 h. Later, the fluorigenic redox indicator dihydroethidium, DHE (HEt; Molecular Probes, ThermoFisher Scientific, Madrid, Spain) was added to the medium and fluorescence was recorded every 15–30 s during a 15 min period, using excitation at 535 nm and emission at 635 nm in a spectrofluorimeter (Bio-Tek FL 600, BioTek Instruments, Madrid, Spain), as described [21]. For every condition, the linear regression of fluorescence data (expressed in arbitrary fluorescence units, AFU) was calculated, and the slopes (a) of the best fitting lines (y = ax) were considered as an index of superoxide production, as described previously [37].

#### 4.2.7. Statistical Analysis

Data have been expressed as mean ± SEM of results from at least three independent experiments of different batches, each performed in triplicate. Statistical comparisons between the different experimental treatments were performed by one-way analysis of variance (ANOVA), which was followed by the Holm-Sidak post-test when the analysis of the variance was significant. A *p* value <0.05 was assumed to be statistically significant. The fitting curves for EC_50_ determinations were carried out according to SigmaPlot v.11 (Systat Software INC., 2012).

### 4.3. Estimation of Lipophilicity as Clog P

Bioloom of Biobyte Corp was used for the theoretical calculation of lipophilicity as Clog *P* values [38].

### 4.4. In Vitro Antioxidant Assays

#### 4.4.1. In Vitro Inhibition of Soybean Lipoxygenase (SLOX)

Stock solutions of the tested nitrones of 10 mM in DMSO were used from which several dilutions were made to succeed in the determination of IC_50_ values. The experimental conditions followed our previously reported protocol [18]. Sodium linoleate was used as a substrate (0.1 mM) with 0.2 mL of the saline solution of SLOX (1/9 × 10^−4^ *w*/*v*) in the Tris buffer pH 9. The enzymatic reaction was recorded spectrophotometrically at 234 nm using NDGA as a reference drug.

#### 4.4.2. Inhibition of Lipid Peroxidation

The reactants consisting of 10 µL (16 mM) sodium linoleate, 10 µL of the tested nitrones in DMSO (final concentration 100 µM), 50 µL of AAPH (40 µM) as a free radical initiator, 930 µL phosphate buffer pH 7.4 (0.05 M) at 37 °C under air, were mixed and the reaction’s result (as absorbance value) was recorded at 234 nm [34] in comparison to PBN as a reference.

#### 4.4.3. Reducing Ability—Interaction to DPPH

The reducing ability of the compounds (50 μM and 100 μM) as absorbance change is measured at rt by their interaction with a solution of DPPH (50 µM) in absolute ethanol after 20 and 60 min at 517 nm [39] with NDGA as reference positive control.

#### 4.4.4. Hydroxy Radicals Scavenging Activity Assay

The competition of the tested nitrones (100 µM) with DMSO (33 mM) in the phosphate buffer (50 mM), pH 7.4, was used [29]. The hydroxyl radicals were generated by the Fe^3+^/ascorbic acid system (EDTA 0.1 mM, Fe^3+^ 167 µM, ascorbic acid 10 mM), and the samples were incubated at 37 °C/30 min. A 17% *w/v* solution of CCl_3_CO_2_H was used to stop the radical propagation. The percentage scavenging activity was compared to Trolox used as a reference.

#### 4.4.5. ABTS^∙+^–Decolorization Assay

Approximately 10 µL of the tested compounds in DMSO (100 µM final concentration) were mixed with 200 µL of ABTS∙^+^ (100 mM) and 790 µL absolute ethanol [34]. The absorbance value was recorded 1 min after mixing at 734 nm and compared to Trolox.

## Figures and Tables

**Table 1:** In vitro antioxidant activity of nitrones **9a**–**i** ^a^.

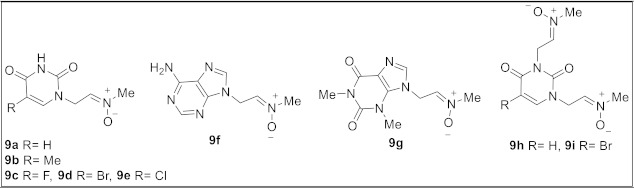						
Standards/Nitrones	Clog *P*	LOX (%)/IC_50_	ILPO (%)	DPPH (%)	OH (%)	ABTS^+^ (%)
**NDGA**		0.45 μΜ		93		
**Trolox**			93		73	91
**PBN**	3.02	23%	11	nt	no	5
**9a**	−2.35	62.5 μΜ	62	20	No	19
**9b**	−1.85	79 μΜ	55	11	73	14
**9c**	−1.87	28 μΜ	59	18	99	5
**9d**	−1.15	35 μΜ	84	6	90	12
**9e**	−1.30	65 μΜ	68	13	87	19
**9f**	−2.09	31 μΜ	88	9	66	6
**9g**	−1.5	33 μΜ	92	15	49	8
**9h**	−1	30 μΜ	No	82	25	50
**9i**	−0.13	57.5 μΜ	71	76	No	75

^a^ Nitrones tested at 100 µM. Values are means ± SD of three or four different determinations. Means within each column differ significantly (*p* < 0.05). nt, not tested.

## Data Availability

The data presented in this study are available on request from the corresponding author.

## Abbreviations

AAPH: 2,2′-Azobis(2-amidinopropane) dihydrochloride; BBB, Blood−brain barrier; BN, bisnitrone; DHE, Dihydroethidium; DPPH, 1,1-Diphenyl-2-picrylhyrazyl radical; HBNs, HomoBisNitrones derived of **PBN**; ILPO, Inhibition of lipid peroxydation; IR, Ischemia reperfusion; LDH, Lactate dehidrogenase; LOX, Lipoxygenase; LP, Lipid peroxidation; **NAC**, N-Acetyl-cysteine; NDGA, Nordihydroguaiaretic acid; OGD, Oxygen-Glucose deprivation; OGDR, Oxygen and Glucose Deprivation and Resupply; OGR, Oxygen and Glucose Resupply; OS, Oxidative stress; PNB, α-phenyl-N-tert-butylnitrone; ROS, Reactive Oxygen Species; SNP Sodium nitroprusside.
